# Contrast Media Induced Kounis Syndrome: A Case Report

**DOI:** 10.3390/diagnostics9040154

**Published:** 2019-10-18

**Authors:** Da-Sen Chien, Andy Po-Yi Tsai, Po-Chen Lin, Giou-Teng Yiang, Meng-Yu Wu

**Affiliations:** 1 Department of Emergency Medicine, Taipei Tzu Chi Hospital, Buddhist Tzu Chi Medical Foundation, New Taipei 231, Taiwan; sam.jan1978@msa.hinet.net (D.-S.C.); taipeitzuchier@gmail.com(P.-C.L.); gtyiang@gmail.com (G.-T.Y.); 2 Department of Emergency Medicine, School of Medicine, Tzu Chi University, Hualien 970, Taiwan; 3 Department of Medical Research, Buddhist Tzu Chi General Hospital, Hualien 970, Taiwan; tandy@iu.edu

**Keywords:** contrast media, Kounis syndrome, cardiac arrest, acute coronary syndrome

## Abstract

Kounis syndrome is a rare anaphylactic reaction leading to coronary spasm, acute plaque rupture, or intrastent stenosis. Many types of medicine or environmental factors can potentially trigger Kounis syndrome by mast cell allergic reactions. In severe Kounis syndrome, reduced blood pressure or cardiac arrest may be accompanying symptoms. The treatment strategy for Kounis syndrome is usually difficult due to both cardiac dysfunction and allergic reactions. The delay to intervention to break the vicious circle may cause catastrophic complications. Therefore, early diagnosis is critical for physicians to outline detailed etiology for prevention and treat the cardiac and allergic symptoms in a timely manner. Here, we describe a case presenting rare severe Kounis syndrome with cardiac arrest which occurred after the administration of a contrast media.

## 1. Introduction

Kounis syndrome is a rare clinical entity that is characterized by anaphylactic or anaphylactoid reaction-induced acute coronary events. A skin reaction is usually detected, and shock status may be the severe accompanying symptom. Several causes have been identified which have the ability to induce Kounis syndrome, including drugs and insect stings. Kounis syndrome consists of three major types: type I—coronary spasm; type II—coronary erosion-induced acute myocardial infarction; and type III—stent thrombosis due to local allergic inflammation. The detailed pathophysiology of Kounis syndrome is still unclear. In the current concept, mast cell activation plays an important role in inducing acute coronary events. Histamine is also an important representative mediator involving these events. Treatment of Kounis syndrome may be difficult due to simultaneously and rapidly occurring cardiac and allergic symptoms, inducing shock status, and cardiac arrest. Therefore, early diagnosis is critical for emergency physicians to outline the etiology of acute coronary events and break the vicious circle of Kounis syndrome. We describe a case presenting rare severe Kounis syndrome secondary to radioiodine contrast. The etiology and clinical features of Kounis syndrome are discussed.

## 2. Case Presentation

**Figure 1:**
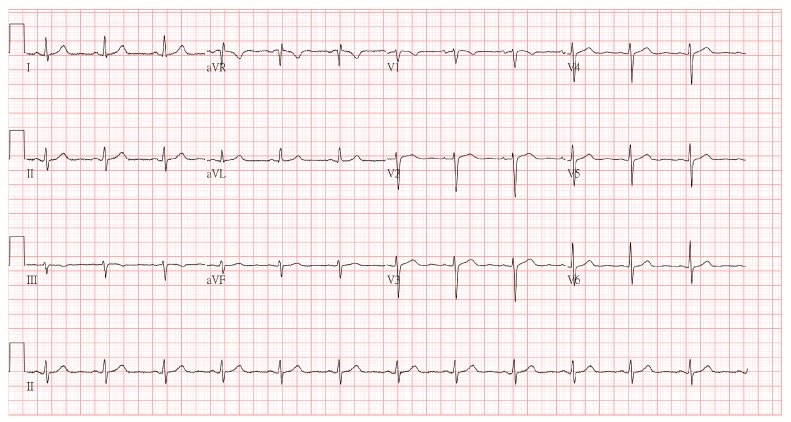
The initial electrocardiogram revealed normal sinus rhythm without any ST segmental elevation or depression.

**Figure 2:**
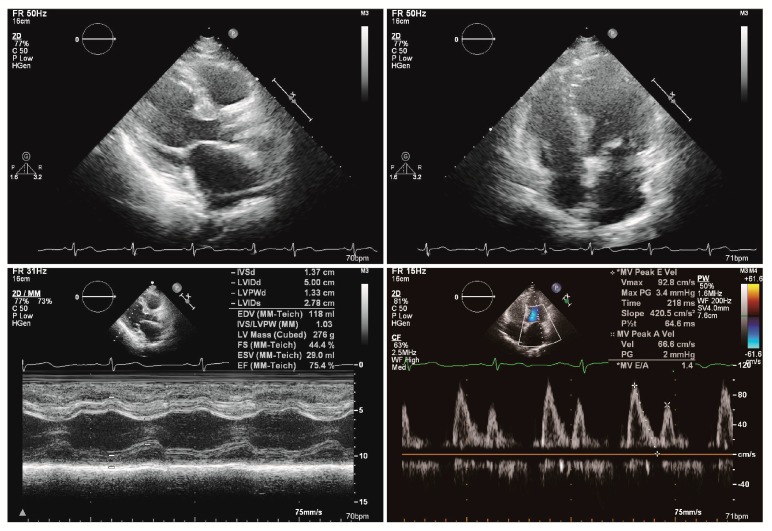
The cardiac ultrasound showed normal wall motion with a left ventricular ejection fraction of 75.4%. There was no significant valvular disorder.

An 81-year-old female presented with progressive epigastric pain experienced over several months. She had a past medical history of breast cancer stage III and had undergone a modified radical mastectomy. Further, she had had acute appendicitis and had an appendectomy, and acute cholecystitis and had a cholecystectomy. The patient has no cardiovascular disease history or family history of cardiovascular disease. Initially, she was suffering from progressive left chest pain for three days. The pain was localized at the left chest without radiation to back, chin, or shoulder. The electrocardiogram (ECG) revealed normal sinus rhythm (Figure 1). The cardiac ultrasound demonstrated a dilated left atrium with adequate left and right ventricular systolic function. Normal wall motion was noted with a left ventricular ejection fraction (LVEF) of 75.4% (Figure 2). Initially, high sensitivity Troponin I was within the normal range. After the administration of a non-steroidal anti-inflammatory drug, she felt better.

However, the chest tightness associated with epigastric pain was noted after three days. She denied diarrhea, tarry stool, bloody stool, vomiting, or nausea. On admission, her temperature was 36.7 °C, blood pressure was 223/102 mmHg, and heart rate was 94 beats/min. On physical examination, an ovoid abdomen was noted with local epigastric tenderness. There was no muscle guarding, Murphy sign, or McBurney’s point tenderness. The laboratory test revealed a high serum level of FDP-Ddimer and no elevation of high sensitive Troponin I. The detail laboratory result is listed in Table 1.

**Figure 3:**
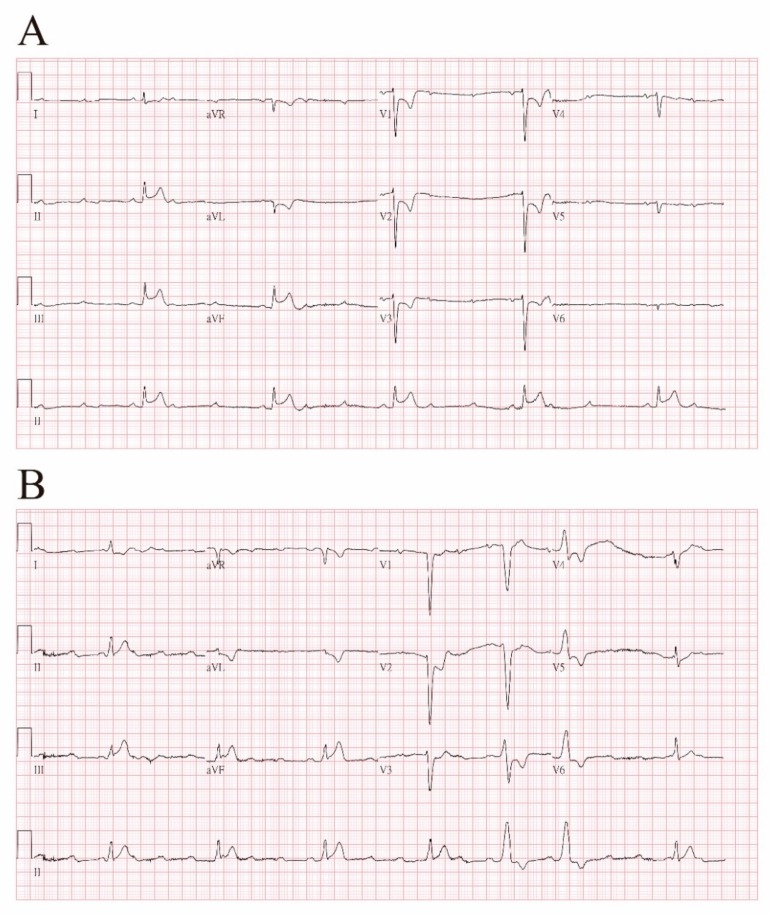
Fifteen minutes after the administration of contrast media, (**A**) ECG showed progressive bradycardia with ST segmental elevation at leads II, III, and aVF, and ST segmental depression at V1 to V6. (**B**) Right side ECG revealed bradycardia with ST segmental change at V2 to V6.

The emergency chest and abdominal contrast-enhanced computed tomography (CT) were done to rule out aorta dissection. However, 15 min after the administration of the contrast media, she lost consciousness, and there was the sudden onset of a whole-body skin rash. The ECG showed progressive bradycardia with ST segmental elevation at leads II, III, and aVF and ST segmental depression at V1 to V6. The right side ECG revealed bradycardia with ST segmental change at V2 to V6 (Figure 3).

**Figure 4:**
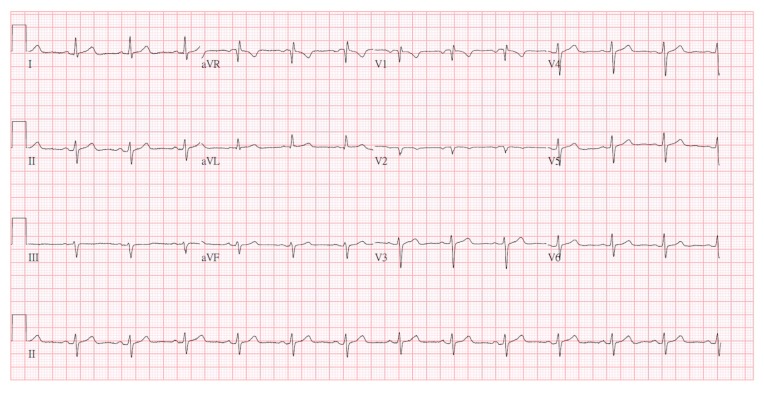
After one month, the follow-up ECG showed no significant ST segmental elevation or depression. The pathologic Q wave was not seen in our patient.

The emergency administration of Atropine 0.5 mg and Betamethasone 4 mg was given. Cardiac arrest with pulseless electrical activity occurred. Cardiopulmonary resuscitation (CPR) was performed immediately. After two minutes with persistent cardiogenic shock, spontaneous circulation (ROSC) returned. Dopamine 10 mcg/kg/min was used to maintain mean arterial pressure (MAP) >65 mmHg. She recovered consciousness with a Glasgow Coma Scale (GCS) score of 11 (E4VTM6). The bedside cardiac ultrasound revealed adequate LVEF without any dyskinesia. At the same time, we also consulted a cardiologist for a differential diagnosis of acute coronary syndrome and to implement early percutaneous coronary intervention (PCI). Although PCI is initially considered as a procedure to diagnose coronary spasm or atherosclerosis, it was suggested that due to the high risk of severe recurrent anaphylactic reactions, it be withheld in our patient. During the PCI procedure, the administration of a contrast media may trigger recurrent anaphylactic shock and, potentially, Kounis syndrome. This patient was admitted to the intensive care unit (ICU) and closely monitored using laboratory tests, ECG, and echocardiography. During hospitalization in the intensive care unit (ICU), there was no chest pain or chest tightness. The follow-up ECG showed the resolution of ST segment elevations. After supportive care, extubation was performed. She was regularly followed up at the outpatient department. After one month, the ECG showed no significant ST segmental elevation or depression. The pathologic Q wave was not seen in our patient (Figure 4). This research has obtained the patient’ consent.

## 3. Discussion

**Figure 5:**
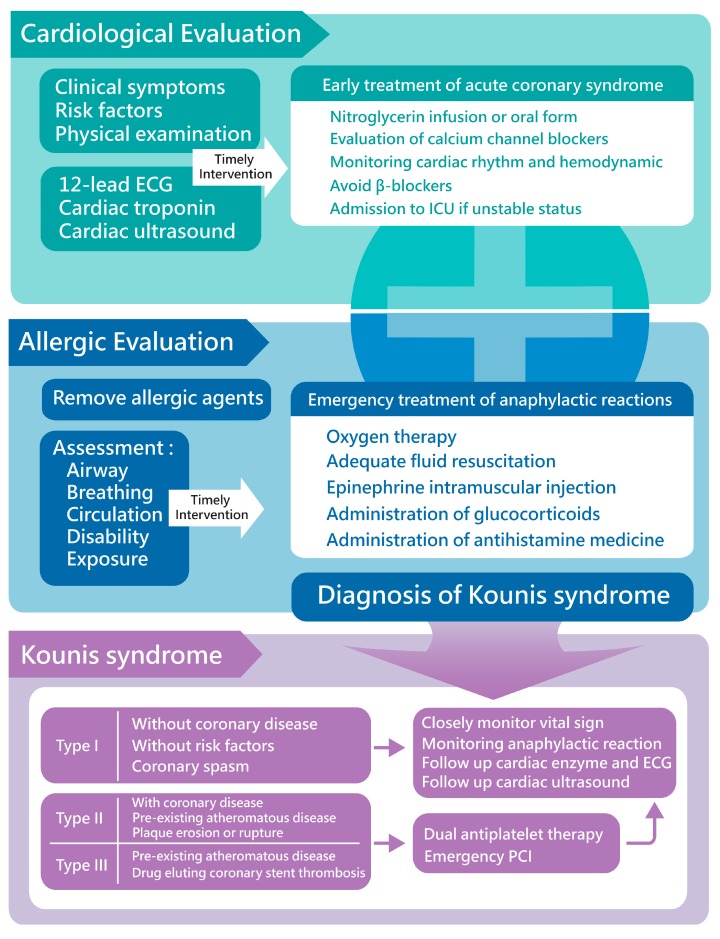
The detailed management of Kounis syndrome.

Kounis syndrome is defined as acute coronary syndromes (ACS) induced by hypersensitivity and anaphylactic reaction. Several causes are reported to induce Kounis syndrome, such as medicines, environmental exposures, bee stings, and asthma (Table 2) [1]. Ayhan Akoz et al. [2] conducted a prospective study and found the incidence of Kounis syndrome in all admissions to be 19.4/100,000. The most common etiology reported was the use of medications, accounting for about 81%. In the current concept, three variants of Kounis syndrome have been described [3]. Type I variant is characterized by allergy-related coronary spasm without coronary lesions or risk factors. Patients with type I Kounis syndrome present electrocardiographic change with or without cardiac enzymes elevation due to the acute release of inflammatory mediators inducing coronary spasm [4]. The type II variant includes pre-existing atheromatous disease. An acute allergic reaction leading to the release of inflammatory mediators induces plaque erosion or rupture, which causes acute myocardial infarction. Type III has been defined in patients with pre-existing coronary disease and coronary artery stent thrombosis [5]. In the Jack P. Chen et al. study, the pathology of drug-eluting stent (DES) thrombosis in these patients presented with eosinophils and mast cell infiltration in Giemsa and hematoxylin–eosin staining [6]. In the Stéphane Cook et al. [7] study, the association with late DES thrombosis and local inflammation in histopathology was reported. In intravascular ultrasound analysis, vessel remodeling was also confirmed. Very late stent thrombosis is recognized as a feature of Kounis syndrome (Figure 5).

The detail pathophysiological mechanisms remain elusive. In the current concept, Kounis syndrome is related to inflammatory cell and mast cell-associated disorders. Local inflammatory cell interactions induce hypersensitivity and anaphylactic results by releasing inflammatory mediators after activation. In the Nicholas G. Kounis et al. [8] summary, Kounis syndrome is a complex multisystem disease accompanied by allergy–hypersensitivity–anaphylaxis. During hypersensitivity, the mast cells and lymphocytes release inflammatory mediators, promoting an allergic reaction via a high serum level of histamine, proteases, arachidonic acid products, and chemokines. These factors cause platelet aggregating and tissue factor expression. The proteases also induce plaque erosion and rupture by activating matrix metalloproteinase (MMP). The downstream mediators induce vasoconstriction and worsen coronary vasospasm.

The diagnosis of Kounis syndrome is based on clinical symptoms and cardiac surveys, including cardiac enzyme, electrocardiographic, echocardiographic, and angiography. The serum level of tryptase and histamine can provide more information about the allergic reaction (Figure 5). Tajda Keber et al. [9] suggested measuring cardiac enzymes in acute allergic reaction patients as necessary to diagnose Kounis syndrome promptly and manage cardiac injury early and appropriately. Echocardiography and coronary angiography can provide detailed information to rule out takotsubo cardiomyopathy or other cardiac disorders in cardiac wall motion abnormalities patients [10]. Keita Goto et al. [11] reported that thallium-201 (Tl) single-photon emission CT (SPECT) and 125I-15-(p-iodophenyl)-3-(R,S)-methylpentadecanoic acid (BMIPP) SPECT were conducted in a post-Kounis syndrome patient, and they revealed a local perfusion defect with decreased BMIPP uptake. Aylin Okur et al. [12] included 26 patients with known or suspected Kounis syndrome and conducted contrast-enhanced magnetic resonance imaging. The results revealed an early-phase subendocardial contrast defect and local edema in lesion areas in T2-weighted images. These newer images provide a reliable study to assess cardiac injury in Kounis syndrome.

Compared to the majority of ACS subjects, the therapeutic strategy of Kounis syndrome should focus on both the cardiac injury and allergic reaction. Sometimes, the two different conditions may require opposing treatments. In the current concept, the administration of medicine should avoid promoting an allergy reaction and aggravating the cardiac injury. Intravenous corticosteroids (hydrocortisone: 1–2 mg/kg/day) and antihistamine agents (diphenhydramine: 1–2 mg/kg) are useful to control the allergic reaction [13]. Aspirin is, however, potentially detrimental because of cyclooxygenase inhibition, promoting arachidonic acid release into the leukotriene pathway [14]. Calcium channel blockers and nitrates can relieve the vasospasm. In hemodynamically unstable patients, nitrates may not be appropriate due to their hypotension properties. Opioids may induce mast cell degeneration, which may worsen the anaphylaxis [15]. Beta-blockers should be used with extreme caution because they can exaggerate coronary spasms and cause epinephrine to be ineffective. Epinephrine is the only life-saving drug in the event of aggravated ischemia, vasospasm, and arrhythmias. In some animal studies, adrenaline may cause LV impairment and not recovery in established anaphylactic shock [16,17]. Therefore, adrenaline is only suggested in high-grade anaphylactic reactions. Adequate fluids resuscitation and oxygen therapy are important supportive treatments for Kounis syndrome. Type II variant patients can take advantage of an ACS protocol. Double antiplatelet therapy in the pharmacological treatment of myocardial revascularization is suggested, including acetylsalicylic acid (ASA) and another P2Y12 receptor inhibitor. In the type III variant, the ACS protocol accompanied by urgent aspiration of intrastent thrombus is suggested (Figure 5). Antihistamines, corticosteroids, and mast cell stabilizers may be helpful for allergic symptoms after stent implantation or revascularization.

The early diagnosis of Kounis syndrome and implementation of an adequate therapeutic strategy is challenging for physicians due to two difficult conditions: the allergic reaction and acute coronary syndrome. In our patient, the allergic reaction was treated with corticosteroids and antihistamines and emergency cardiac evaluation. An acute coronary event protocol was considered due to cardiac arrest with ROSC. However, we finally held the protocol due to adequate LVEF with normal cardiac enzymes and improved ECG findings during ICU hospitalization. We presented this rare case of Kounis syndrome caused by the administration of contrast media. This syndrome should be considered by practitioners of the contrast CT scan. Early management can prevent catastrophic complications in clinical prognosis.

## Figures and Tables

**Table 1:** The laboratory evaluation in this patient.

Variables	Normal Range	Patient Data	Variables	Normal Range	Patient Data
White cell count	3.5–11 × 10^9^/L	9.06	Blood urine nitrogen	2.5–6.4 mmole/L	3.93
Band form neutrophils	0%–3%	5.0	Creatinine	0.04–0.09 mmole/L	0.0972
Segment form neutrophils	45%–70%	51.0	Sodium	136–145 mmole/L	135
Lymphocytes	25%–40%	30.0	Potassium	3.5–5.1 mmole/L	4.7
Eosinophils	1%–3%	1.0	Calcium	2.12–2.52 mmole/L	2.43
Monocytes	2%–8%	1.0	Glucose	3.9–5.6 mmole/L	13.653
Basophils	0%–1%	1.0	Alanine aminotransferase	0.27–1.05 µkat/L	0.6
Hemoglobin	7.45–9.93 mmole/L	9.4	Lipase	73–393 IU/L	293
Platelet counts	150–400 × 10^9^/L	104	Creatine kinase	26–192 IU/L	136
Prothrombin time	8.0–12.0 sec.	11.1	High-sensitive Troponin I	0–19 ng/L	<1.5
Partial thromboplastin time	23.9–35.5 sec.	30.7			
FDP-Ddimer	0–500 µg/L	4930.88			

**Table 2:** Previously reported causes of Kounis syndrome.

Substance	
Medicine	
Antibiotics	Ampicillin, amoxicillin, amikacin, cefazolin, cefoxitin, cerufoxime, cephradine, cinoxacin, lincomycin, penicillin, sulbactam/cefoperazone, piperacillin/tazobactam, trimethoprim/sulfamethoxazole, sulperazon, vancomycin
Anesthetics	Etomidate, isoflurane, midazolam, propofol, remifentanil, rocuronium bromide, succinylcholine, suxamethonium, trimethaphan
Antineoplastics	5-fluorouracil, capecitabine, carboplatin, denileukin, interferons, paclitaxel, vinca alkaloids
Anticoagulants	Heparin, lepirudin
Others	Glucocorticoids, contrast media, nonsteroidal anti-inflammatory drugs, Proton pump inhibitors, skin disinfectants, thrombolytics
Condition	Angioedema, asthma, Churg–Strauss syndrome, hay fever, idiopathic anaphylaxis, intracoronary stenting, mastocytosis, nicotine, Scombroid syndrome, serum sickness
Food	Canned food, egg, milk, fish, shellfish, mushroom, vegetables, tomato, Actinidia chinensis
Environment	Viper venom, insect bites, octopus bite, jellyfish stings, scorpion sting, animal licking, grass cutting, poison ivy, latex contact, metals or millet allergy
